# Cross-population proteome-wide mendelian randomization study identifies likely causal proteins for cardiovascular diseases

**DOI:** 10.1007/s00438-026-02458-4

**Published:** 2026-08-03

**Authors:** Hua Zhong, Jingjing Zhu, Shuai Liu, Hoi Tung Hilton Wong, Yiqiang Zhang, Hung N. Luu, Qing Wu, Xuexia Wang, Lang Wu

**Affiliations:** 1 Department of Interdisciplinary Oncology, LSU-LCMC Health Cancer Center, School of Medicine, Louisiana State University Health Sciences Center, New Orleans, LA USA; 2 Department of Genetics, LSU-LCMC Health Cancer Center, School of Medicine, Louisiana State University Health Sciences Center, New Orleans, LA USA; 3 Cancer Epidemiology Division, Population Sciences in the Pacific Program, University of Hawai’i Cancer Center, University of Hawai’i at Mānoa, Honolulu, HI USA; 4 Department of Quantitative Health Sciences, John A. Burns School of Medicine, University of Hawai’i at Mānoa, Honolulu, HI USA; 5 Biochemistry Program, Department of Chemistry, Colleges of Arts and Sciences, University of Hawai’i at Mānoa, Honolulu, HI USA; 6 Department of Anatomy, Biochemistry and Physiology, John A. Burns School of Medicine, University of Hawai’i at Mānoa, Honolulu, HI USA; 7 Dr. Mary and Ron Neal Cancer Center, Houston Methodist Research Institute, Houston, TX USA; 8 Department of Biomedical Informatics, College of Medicine, The Ohio State University, Columbus, OH USA; 9 Department of Biostatistics, Florida International University, Miami, FL USA

**Keywords:** Proteome, Two-sample Mendelian randomization, Cardiovascular diseases, Different populations, Drug targets

## Abstract

Blood proteins may play causal roles in cardiovascular diseases (CVDs) such as heart failure (HF) and peripheral artery disease (PAD). Proteome-wide Mendelian randomization (MR) has been widely used to prioritize drug targets for CVD in European populations, but its application to non-European populations remains limited. We conducted a proteome-wide MR analysis to evaluate the potential causal effects of 2,922 plasma proteins on five CVDs—atrial fibrillation (AF), coronary artery disease (CAD), HF, ischemic heart disease (IHD), and PAD. Analyses were performed across African (n = 931), East Asian (n = 262), and European (n = 10,840) populations using genetic instrument data from the UK Biobank cohort. Significant associations were further examined with genetic colocalization to strengthen causal inference. Using MR and colocalization analyses, we identified 53 significant protein–CVD associations across multi-populations, including 16 in African, six in East Asian, and 31 in European populations, respectively. Cross-population comparisons revealed four protein–CVD associations unique to African population and another four specific to East Asian population. Integration with clinical trial data prioritized 14 protein–disease pairs as promising candidates for therapeutic development or drug repurposing. Our findings highlight the value of proteome-wide MR in evaluating drug target applicability across populations. Several protein–disease associations were population-specific, emphasizing the need for inclusive genetic research to inform precision medicine in CVD prevention and treatment.

**Supplementary Information:**

The online version contains supplementary material available at 10.1007/s00438-026-02458-4.

## Introduction

Cardiovascular diseases (CVDs) remain the leading cause of mortality and a major contributor to disability worldwide (Joseph et al. 2017). Identifying causal relationship between circulating protein biomarkers and CVD outcomes is crucial for informing treatment regimens and therapeutic development. Traditional epidemiologic studies have identified numerous potential protein biomarkers for CVD. However, their interpretation is often limited by selection bias, residual confounding, and reverse causation (Zhong et al. 2023a, b). These issues hinder the translation of observational findings into reliable clinical applications and the identification of effective therapeutic targets.

Mendelian randomization (MR) is an epidemiological method that can be used to obtain evidence about the causal effects of modifying intervention targets (Richmond and Smith 2022), which has become increasingly popular in chronic disease research. Using genetic variations that are randomly assigned at conception as proxies for exposure to a risk factor, MR approach mitigates biases common in traditional observational study designs, such as unmeasured confounding and reverse causality (Levin and Burgess 2024). It also offers an effective quasi-experimental approach that mimics a randomized clinical trial, which is often resource-intensive and expensive (Burgess et al. 2023a; Wouters et al. 2009; Moore et al. 2015). Proteome-wide MR utilizes genetically-associated protein levels to test the putative causal effects of proteins on common diseases (Zhao et al. 2022). By providing insights into protein-mediated disease mechanisms and therapeutic opportunities, proteome-wide MR has become a powerful and accessible tool for guiding drug discovery and development. Recent proteome studies suggest that proteins with robust MR and colocalization evidence are more likely to become successful targets in drug trials (Burgess et al. 2023a; Zheng et al. 2020). Notable examples relevant to CVDs include cholesteryl ester transfer protein (CETP), interleukin 6 receptor (IL6R), N-terminal Niemann–Pick C1 like intracellular cholesterol transporter 1 (NPC1L1), and proprotein convertase subtilisin/kexin type 9 (PCSK9), which are supported by both MR analyses and evidence from phase II and III clinical trials (Burgess et al. 2023a). For example, genetic variations in the *CETP* gene associated with reduced CETP activity provide strong evidence for the protective effect of CETP inhibition on CVD risk (Johannsen et al. 2012). In a phase III trial, the use of anacetrapib, a potent CETP inhibitor, was reported to result in a lower incidence of major coronary events compared with placebo (Bowman et al. 2018).

Despite these advences, genetic association studies have predominantly focused on individuals of European population (McPherson 2014; Sirugo et al. 2019), leading to their overrepresentation in MR studies and limiting the generalizability of biomarker-disease associations to other populations (Zheng et al. 2017). This limitation is particularly relevant for CVDs, where disease incidence, risk factor, and clinical outcomes vary substantially across populations. For example, a national U.S. survey from 1999 to 2018 found that non-Hispanic Black individuals experienced consistently higher age- and sex-adjusted 10-year risk of atherosclerotic CVD than non-Hispanic White individuals (He et al. 1999). Given that circulating plasma proteins are key mediators of cardiovascular function and frequent therapeutic targets (Katz et al. 2022), robust evaluation of protein–CVD relationships across different genetic backgrounds is essential.

Recent multi-population proteome-wide MR studies have begun to address this gap. Zhao et al. conducted a multi-population proteome-wide MR analysis leveraging large-scale biobank meta-analyses to assess the generalizability of protein–disease associations, primarily across African and European ancestries (Zhao et al. 2022). While these efforts provide an important foundation, they are limited by relatively limited phenotypic resolution for cardiovascular diseases, incomplete evaluation of population-specific genetic architecture, and insufficient biological interpretation of population heterogeneity.

To address these limitations, in this study we conducted a large-scale proteome-wide MR investigation of the causal effects of circulating proteins on five major CVDs, including atrial fibrillation (AF), coronary artery disease (CAD), heart failure (HF), ischaemic heart disease (IHD), and peripheral artery disease (PAD) across African, East Asian, and European populations (He et al. 1999). We systematically estimated the putative causal role of 2,922 circulating proteins using a population-specific MR framework that incorporates population-specific protein quantitative trait loci (pQTLs) instruments, cross-population consistency and heterogeneity assessment, and integration with genetic colocalization, pathway enrichment, and clinical trial evidence (Munafò and Davey Smith 2018). This approach extends prior proteome-wide MR efforts by enabling disease-specific, biologically informed, and translationally relevant interpretation of protein–CVD associations across multiple populations.

## Materials and methods

### Instrumental variables for plasma proteins

**Fig. 1:**
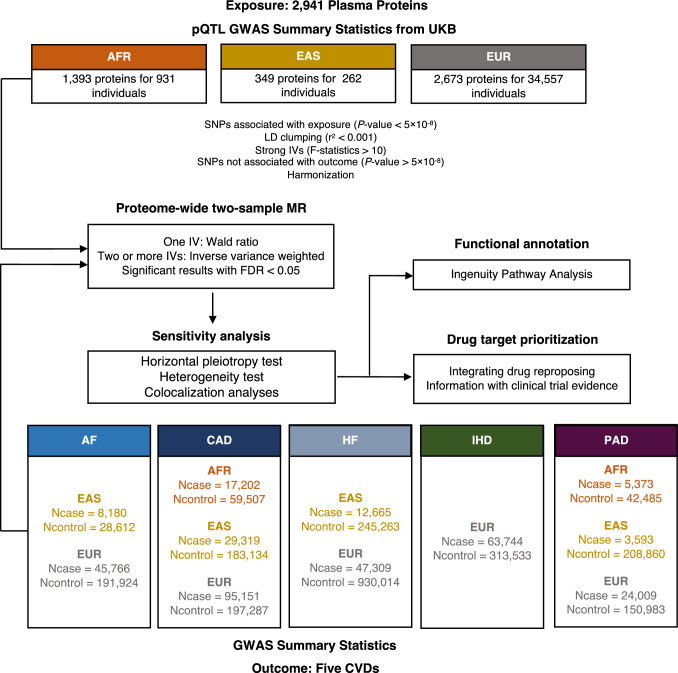
The overall study workflow

Figure 1 provides the framework of the statistical analyses for this study. We selected genetic instruments for 2,922 plasma proteins based on a genome-wide association study (GWAS) of 931, 262, and 34,557 participants of African, East Asian, and European populations, respectively, within the UK Biobank Pharma Plasma Proteome (UKB-PPP) cohort (Sun et al. 2023). Details of UKB participant selection and sample handling have been provided elsewhere (Sun et al. 2023). Proteomic profiling on blood plasma was performed with the Olink Explore 3,072 platform, which measures 2,941 protein analytes representing 2,923 unique proteins. Details of the Olink proteomics assay, data processing, and quality control have been described elsewhere (Sun et al. 2023). Each protein level was inverse-rank normalized before analyses and association testing. M apping was performed for pQTLs using up to 16.1 million imputed variants for 2,922 proteins after quality control.

In this study, 1,952 independent pQTLs of 1,393 proteins in individuals of African population, 357 independent pQTLs of 349 proteins in individuals of East Asian population, and 33,818 independent pQTLs of 2,673 proteins in individuals of European population derived from the UKB-PPP study were selected as candidate genetic instruments. Genetic variants that meet the following criteria were selected as instrumental variables (IVs) (Fig. 1): (1) SNPs associated with the exposure were required to meet a genome-wide significance threshold with a *P*-value < 5 × 10^–8^ (Ghoneim et al. 2020; Wu et al. 2020; Shu et al. 2019); (2) SNPs were filtered using linkage disequilibrium (LD)-based clumping with r^2^ < 0.001 within a window of 10Mb; (3) Alleles were harmonized between exposure and outcome datasets; (4) The IVs should not be directly associated with the outcome (*P*-value > 5 × 10^–8^); and (5) IVs must have an *F*-statistic of at least 10. Both *cis*- and *trans*-pQTLs reaching genome-wide significance and passing instrument strength criteria were included as IVs. After filtering, a summary table displaying the distribution of IVs per protein for each population are provided in Supplementary Table S1.

### GWAS datasets of CVD risk

For CVD outcomes, we compiled genetic association information from several biobank cohorts: Biobank Japan (BBJ) (Sakaue et al. 2021), FinnGen (Kurki et al. 2023), Global Biobank Meta-analysis Initiative (GBMI) (Zhou et al. 2022), and Million Veteran Program (MVP) (Gaziano et al. 2016) cohorts (Supplementary Table S2). Detailed information about the samples collection, data processing, and statistical analyses for these cohorts is available in prior publications (Sakaue et al. 2021; Kurki et al. 2023; Zhou et al. 2022; Gaziano et al. 2016). For AF, we assembled GWAS data of 36,792 East Asian samples (8,180 cases and 28,612 controls) from BBJ cohort and 237,690 European samples (45,766 cases and 191,924 controls) from FinnnGen cohort. GWAS data of CAD were obtained from 76,709 African samples (17,202 cases and 59,507 controls), 292,438 European samples (95,151 cases and 197,287 controls) from MVP cohort, and 212,453 East Asian samples (29,319 cases and 183,134 controls) from BBJ cohort. We included 12,665 HF cases and 245,263 controls of East Asian population (257,928 samples in total) from GBMI cohort and 43,344 HF cases and 258,943 controls of European population (302,287 samples in total) from MVP cohort. For IHD, we gathered GWAS data from 377,277 European samples (63,744 cases and 313,533 controls) from FinnGen cohort. We also compiled GWAS data of PAD from 47,858 African samples (5373 cases and 42,485 controls), 174,992 European samples (24,009 cases and 150,983 controls) from MVP cohort, and 212,453 East Asian samples (3,593 cases and 208,860 controls) from BBJ cohort (Supplementary Table S2).

### Two-sample MR analysis for potential causal associations between protein levels and CVD risk

To test the potential causal effects of proteins on five CVDs within each population, we performed two-sample MR analyses using the “TwoSampleMR” R package (Hemani et al. 2018). SNPs were aligned based on effect and non-effect allele information to ensure consistent orientation across pQTL and GWAS datasets. Palindromic variants (A/T or C/G) were resolved using allele frequency information where available, and variants with unresolved strand ambiguity were excluded (Burgess et al. 2023b). All variants were mapped to the GRCh37 (hg19) reference genome. For each protein, we selected the pQTL with the lowest *P*-value as the primary IV to improve instrument specificity and reduce redundancy. For proteins with a single available pQTL, causal effects were estimated using the Wald ratio test (Lawlor et al. 2008). For proteins with multiple IVs, we employed inverse variance weighted (IVW) analysis under a fixed-effects model (Burgess et al. 2017; Zheng et al. 2021). Odds ratio (OR) and 95% confidence interval (CI) were calculated to quantify the associations between IVs and CVD risk. Statistical significance was determined at a false discovery rate (FDR) < 0.05 for each disease within each population.

### Sensitivity analyses of candidate MR signals

To test the robustness of MR assumptions and identify potential sources of bias, we performed a set of sensitivity analyses for the candidate MR signals. First, the MR-Egger method (Bowden et al. 2015) and MR pleiotropy residual sum and outlier (MR-PRESSO) global test (Verbanck et al. 2018; Wu et al. 2021) were used to evaluate horizontal pleiotropy. Evidence of directional pleiotropy was considered significant if *P*_Egger-Intercept_< 0.05 or *P*_GlobalTest_< 0.05. The potential outlier SNPs were identified using the MR-PRESSO outlier test.The corrected *P*-value was calculated after excluding the outliers detected by the MR-PRESSO method. Second, we conducted Bayesian colocalization analysis using the 'coloc' R package (Giambartolomei et al. 2014). A posterior probability (PP.H4) > 70% was set as the threshold, indicating that the genetic association signals for both the protein and the phenotype are likely influenced by the same causal variant. The GWAS-pQTL colocalization events with the highest PP.H4 were visualized using the ‘LocusCompareR’ R package (Liu et al. 2019). Third, we performed meta-analyses of population-specific association results for each CVD disease using an inverse-variance-weighted fixed-effect method. Between-population heterogeneity was assessed using the I^2^ statistic in METAL, with high heterogeneity defined as I^2^ > 75%.

### Ingenuity pathway analysis (IPA)

The IPA was performed to assess enriched pathways, networks, and molecular functions of the genes encoding CVD-associated proteins. The detailed methodology of this tool has been described elsewhere (Krämer et al. 2014). In brief, enrichment significance was evaluated using Fisher’s exact test by assessing overlap between observed gene sets and curated biological pathways. Pathways and molecular functions with enrichment P-values <0.05 were considered statistically significant.

### Drug target prioritizing

In search of potential drug repositioning opportunities, we conducted a thorough exploration of the DrugBank database (Wishart et al. 2006) to identify existing drugs targeting MR-prioritized proteins. For proteins with available drug-target evidence, relevant clinical trial information was further evaluated using ClinicalTrials.gov (https://clinicaltrials.gov/).

## Results

### Identification of significant protein-disease pairs across populations

**Fig. 2:**
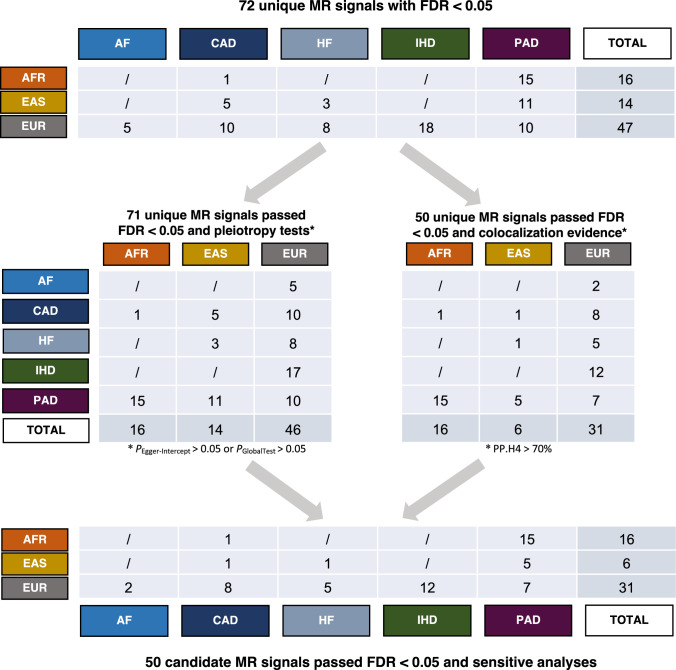
The statistics of protein–CVD associations identified by MR and sensitivity analyses. *AFR* Africans, *EAS* East Asians, *EUR* Europeans, *AF* atrial fibrillation, *CAD* coronary artery disease, *HF* heart failure, *IHD* ischemic heart disease, *PAD* peripheral artery disease, *CVD* cardiovascular disease, *MR* Mendelian randomization, *FDR* false discovery rate, *PP.H4* a posterior probability in Bayesian colocalization analysis

Out of 1360, 306, and 2611 unique protein-CVD pairs analyzed in African, East Asian, and European populations, respectively, we identified 16, 14, and 47 significant MR signals that reached an FDR < 0.05 for at least one CVD in the corresponding populations. In the African population, one protein was significantly associated with CAD risk, and 15 proteins were significantly associated with PAD risk (Fig. 2). In the East Asian population, we also observed five significant protein-CAD risk associations, three protein-HF risk associations, and 11 significant protein-PAD risk associations. Additionally, in the European population, five, ten, eight, 18, and ten proteins showed significant associations with AF, CAD, HF, IHD, and PAD risk, respectively (Fig. 2).

### Estimation of horizontal pleiotropy of MR signals

Horizontal pleiotropy occurs when a genetic variant affects disease through pathways other than the exposure, violating a key assumption of MR. While using multiple genetic variants can enhance statistical power, it may also increase the possibility of pleiotropic effects. To assess this, we conducted the MR-Egger and MR-PRESSO sensitivity analyses for proteins that passed the FDR threshold for associations with CVD risk. With the exception of LDLR for IHD in the European population (*P*_Egger-Intercept_ = 0.037 and *P*_GlobalTest_ < 0.001), all other proteins showed minimal evidence of horizontal pleiotropy, with *P*_Egger-Intercept_ > 0.05 or *P*_GlobalTest_ > 0.05. For LDLR, MR-PRESSO identified four outlier SNPs, which were removed individually rather than en masse, and the MR estimate was recalculated. After outlier removal, the association remained significant (*P*_Outlier-Corrected_ = 3.02 × 10^–3^), indicating that the causal estimate was robust to pleiotropy (Supplementary Table S3). Overall, these results suggested that none of the identified protein-CVD associations in African, East Asian, or European population showed evidence of being influenced by horizontal pleiotropy (Supplementary Table S3).

### Colocalization analyses

To further prioritize putative causal proteins for CVD rather than mere linkage, we conducted Bayesian colocalization analysis. This approach revealed colocalization evidence for two MR signals for AF in Europeans, ten MR signals for CAD (including one in Africans, one in East Asians, and eight in Europeans), six MR signals for HF (one in East Asians and five in Europeans), 12 MR signals for IHD in Europeans, and 26 MR signals for PAD comprising 15 in Africans, five in East Asians, and seven in Europeans (Supplementary Table S3).

As shown in Supplementary Table S3, a total of 53 MR signals involving 50 unique proteins, including 16 in Africans, six in East Asians, and 31 in Europeans, surpassed the FDR-corrected threshold and showed colocalization evidence with minimal indications of pleiotropy.

### Functional annotation by IPA

We performed an IPA for the 50 CVD-associated proteins that were identified through robust MR and colocalization with minimal pleiotropy in at least one population. The analysis revealed key canonical pathways, including plasma lipoprotein assembly, remodeling, and clearance (*P*-value = 5.00 × 10^−7^), HEY1 signaling pathway (*P*-value = 2.01 × 10^−5^), IL-17 signaling (*P*-value = 4.12 × 10^−5^), and Notch signaling (*P*-value = 6.59 × 10^−5^) (Supplementary Table S4). Several CVD-related functions and canonical pathways (i.e., regulation of the epithelial-mesenchymal transition pathway) were significantly enriched for the genes encoding proteins linked to AF risk (*FGF5* and *TGFB2*) at *P*-value < 0.05. For genes encoding ten proteins associated with CAD, the most significant enriched canonical pathway was the Regulation of Insulin-like Growth Factor (IGF) transport and uptake by IGFBPs (*P*-value of 1.14 × 10⁻^3^). Among genes encoding six proteins linked to HF, significantly enriched CVD-related canonical pathways included cachexia signaling pathway (*P*-value = 6.77 × 10⁻^5^), transcriptional regulation of white adipocyte differentiation (*P*-value = 1.77 × 10⁻^4^), and atherosclerosis signaling (*P*-value = 4.44 × 10⁻^4^). For genes encoding 12 proteins associated with IHD, the top enriched canonical pathways involved plasma lipoprotein assembly, remodeling, and clearance (*P*-value = 4.38 × 10⁻⁸), LXR/RXR activation (*P*-value = 2.74 × 10⁻^5^), retinoid metabolism and transport (*P*-value = 2.11 × 10⁻^4^), atherosclerosis signaling (*P*-value = 1.91 × 10⁻^3^), and nerve growth factor (NGF) processing (*P*-value = 1.98 × 10⁻^3^). Additionally, for genes encoding 27 proteins associated with PAD, the primary enriched canonical pathways included HEY1 signaling (*P*-value = 6.66 × 10⁻^4^), notch signaling (*P*-value = 7.57 × 10⁻^4^), and NOTCH3 signaling (*P*-value = 1.31 × 10⁻^3^) (Supplementary Table S4).

### Identification of pan-population and population-specific associations with heterogeneity assessment

**Fig. 3:**
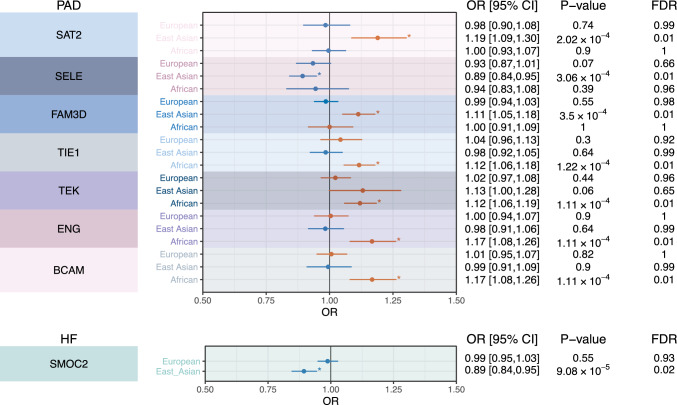
Significant protein-CVD associations with MR and colocalization evidence. Forest plots showing Mendelian randomization (MR) estimates for significant protein–cardiovascular disease (CVD) associations identified in African, East Asian, and European populations. Effect sizes are presented as odds ratios (ORs) with 95% confidence intervals (CIs) per genetically predicted standard deviation increase in protein levels. Associations shown passed multiple-testing correction and colocalization analysis

**Fig. 4:**
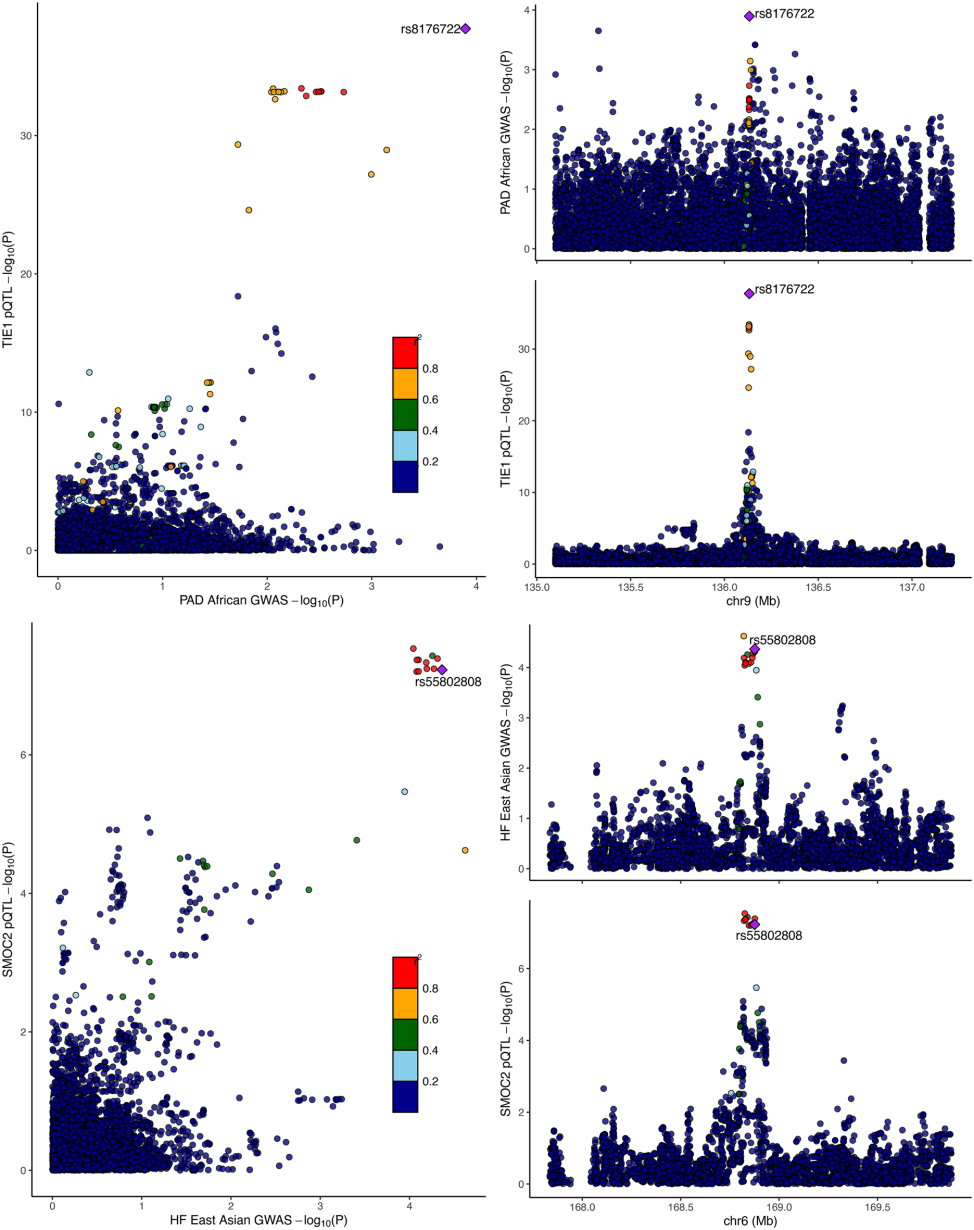
Visualization of GWAS-pQTL colocalization. GWAS-pQTL colocalization events for candidate proteins associated with CVD risk exhibiting the highest posterior probability of colocalization (PP.H4) were shown. The left panel displays correlation plots comparing GWAS and pQTL association signals, while the right panel presents regional association plots for the same loci. The labeled SNP represents the lead variant for both the GWAS and pQTL analyses. All other SNPs are colored according to their linkage disequilibrium (LD; r^2^) with the lead SNP, illustrating the local correlation structure of the genomic region. *CVD* cardiovascular disease, *GWAS* genome-wide association study, *HF* heart failure, *pQTL* protein quantitative trait loci, *PAD* peripheral artery disease, *SNP* single-nucleotide polymorphism

We conducted a cross-population comparison of unique protein-disease association pairs to identify both shared and population-specific MR signals. Applying an FDR threshold of 0.05 to the 50 protein-CVD pairs, we identified VWF as a protein strongly associated with PAD, supported by robust MR and colocalization evidence in both African (FDR = 0.01, PP.H4 = 94.2%) and European (FDR = 0.04, PP.H4 = 93.1%) populations. Additionally, we identified population-specific protein-CVD associations that exhibited causal effects in one population (FDR < 0.05) but not in others (*P*-value > 0.05). For example, SMOC2 was identified as a protein specifically associated with HF in the East Asian population, supported by MR evidence (FDR = 0.02 in Asian population; *P*-value = 0.55 in European population) and colocalization evidence (PP.H4 = 92.5%) (Fig. 3). Furthermore, we identified four African-specific proteins (BCAM, ENG, TEK, and TIE1) and three East Asian-specific proteins (FAM3D, SAT2, and SELE) associated with PAD, all supported by MR and colocalization evidence (Figs. 3, 4, Supplementary Table S3).

To further evaluate heterogeneity, we meta-analyzed data for AF, CAD, HF, and PAD across populations using METAL (Willer et al. 2010). IHD was excluded from this analysis as its associations were only evaluated in the European population. Based on the criterion of an I-square (HetISq) > 75%, we identified four proteins (CLEC4M, SERPIND1, ABO, and CD209) showing heterogeneity for CAD and two proteins (SELE and SMOC2) for HF. Notably, among the 35 proteins associated with PAD, 20 demonstrated significant heterogeneity, suggesting substantial variation across populations (Supplementary Table S5).

### Drug target prioritization

Of the 50 identified CVD-associated proteins with colocalization evidence and minimal pleiotropy in at least one population, 14 were annotated as targets of currently approved drugs for various human diseases. For example, cadherin-5 (CDH5) has been annotated as a target of Lenalidomide (Drugbank ID DB00480), which is used to treat multiple myeloma (Zhang et al. 2024). Our study identified a potential causal role of CDH5 levels in PAD risk, suggesting a repurposing opportunity of Lenalidomide for PAD treatment. These findings underscore the potential for repurposing these drugs to treat CVDs (Supplementary Table S6). Additionally, we investigated clinical trial data for these 14 prioritized proteins using the ClinicalTrials.gov database (Supplementary Table S7). Among them, we identified that Carvedilol (Drugbank ID DB01136), annotated as an E-selectin inhibitor in DrugBank, had been approved for HF treatment (Packer et al. 2002). This aligns with our MR findings, which support the potential involvement of SELE in HF risk (Supplementary Table S7).

## Discussion

Our analysis identified a total of 50 unique proteins associated with the risk of at least one CVD based on MR and genetic colocalization evidence, including 16 proteins in African, six in East Asian, and 31 in European populations (Supplementary Table S3). Among them, 36 proteins or their protein-encoding genes had previously been implicated to be associated with CVD outcomes or mapped to canonical cardiovascular GWAS loci. For example, proprotein convertase subtilisin/kexin type 9 (PCSK9), a key regulator of lipid metabolism, has been implicated in CAD development (Gai et al. 2021). PCSK9 increases plasma cholesterol by promoting the degradation of low-density lipoprotein receptor (LDLR) (Lambert et al. 2012). Elevated PCSK9 expression is associated with higher LDL cholesterol levels and poorer cardiovascular prognosis (Panahi et al. 2019), while gain-of-function variants in *PCSK9* are linked to hypercholesterolemia and increased CAD risk (Cohen et al. 2005, 2006). In our study, we confirmed positive associations between predicted PCSK9 levels and CAD risk in Europeans. We also identified fibroblast growth factor 5 (FGF5) to be associated with AF risk, supporting its potential as a novel therapeutic target for AF (Supplementary Table S3).

Several well-established CVD biomarkers (Rayat et al. 2022; Kamstrup and Nordestgaard 2016; Theis et al. 2022; Kozlov et al. 2022; Zhang et al. 2012; Roldán et al. 2003) were also identified to be associated with the risk of at least one CVD in our study. For instance, ABO was linked to the risks of CAD, IHD, and PAD, while CELSR2, LPA, SMOC2, and SELE were associated with risks of both PAD and HF (Supplementary Table S3). These findings are consistent with previous genetic epidemiologic studies (Alanne 2008; Chen et al. 2024; Whincup et al. 2002; Castillo-Avila et al. 2023; He et al. 2012) and further support the critical roles of these biomarkers in cardiovascular pathology. Additionally, our results underscore the shared pathophysiological mechanisms underlying CAD, PAD, HF and IHD, emphasizing the complex interplay among these atherothrombotic CVDs. Although many of these loci have been robustly validated over the past decade (Kamstrup and Nordestgaard 2016; Whincup et al. 2002; Castillo-Avila et al. 2023; He et al. 2012), our cross-ancestry framework reveals ancestry-specific effect sizes and heterogeneity that were previously poorly recognized. For example, ABO blood group genotypes are well-established causal risk factors for CVDs (Zhang et al. 2012; He et al. 2012; Sari et al. 2008). We found that ABO was associated with PAD risk in Africans and with CAD risk in East Asians (Supplementary Table S3). A pilot study suggested that blood group A might be associated with higher CAD risk in African population compared with non-A blood groups (Ba et al. 2017). In the Japanese population, Matsunaga et al. identified 19 CAD-associated loci through two GWAS, including 9q34 (*ABO*) locus (Matsunaga et al. 2020). These findings align well with our results and underscore the importance of the ABO locus in influencing CVD risk in non-European populations. Furthermore, our MR analysis suggested potential heterogeneity across populations in the associations of eight protein-CVD pairs. For instance, BCAM protein levels were likely causally associated with PAD in Africans (*P*-value = 1.11 × 10⁻^4^), but not in European (*P*-value = 0.82) or East Asian (*P*-value = 0.90) populations (Fig. 3 and Supplementary Table S3), highlighting ancestry-specific biological effects.

Pathway enrichment analysis revealed key biological mechanisms underlying the observed protein–CVD associations, with PAD-associated genes significantly enriched in Notch and HEY1 signaling pathways. Previous genetic and in vitro studies have demonstrated that Notch signaling regulates macrophage activation and contributes to cardiovascular calcification (Rusanescu et al. 2008). In particular, activation of NOTCH3 induces downstream transcription factors *HEY1* and *HEY2* in vascular smooth muscle cells (Fischer and Gessler 2003; Wang et al. 2008; Boucher et al. 2012), linking Notch signaling to vascular remodeling. Collectively, these findings support a role for dysregulated Notch–HEY signaling in impaired endothelial function and pathological vascular remodeling in PAD pathogenesis.

Beyond these established loci, our integration of plasma proteomics and genetic colocalization identified 14 proteins with limited prior cardiovascular annotation, including ASS1B, CAM, CCAR2, CUZD1, DDX25, DEFB103A, KRT8, MEP1A, MPRIP, PITHD1, PLXDC2, SAT2, SCIN, and THSD1. Several of these proteins have been implicated in non-cardiovascular diseases. For instance, CUZD1 and anti-CUZD1 antibodies have been associated with cancer and inflammatory bowel diseases (Liaskos et al. 2013). Rare variants in *CCAR2* were linked to diabetes risk in the UK Biobank (OR = 13, *P*-value = 8.5 × 10⁻⁸), based on a large-scale rare variant association study (Jurgens et al. 2020). Notably, we identified 14 proteins that are targets of existing drugs (Supplementary Table S6), several of which are already approved for CVD management (Ascef et al. 2016; Kosmas et al. 2020; Li et al. 2016; Papadia et al. 2018; Coccheri and Mannello 2013). For instance, inclisiran (Drugbank ID DB14901), a small interfering RNA therapy targeting PCSK9 (German and Shapiro 2020), is used as an adjunct therapy for atherosclerotic CVDs (Kosmas et al. 2020). Tenecteplase (Drugbank ID DB00031), a thrombolytic agent, is commonly administered for acute myocardial infarction (Ascef et al. 2016). Other drugs, originally developed for non-CVD indications, highlight opportunities but also challenges for drug repurposing. CDH5, which showed a significant association with PAD risk in our MR analysis (*P*-value = 1.11 × 10⁻^5^), has been reported as a lenalidomide-associated target in DrugBank, an immunomodulatory agent approved for multiple myeloma (Zhang et al. 2024) and myelodysplastic syndromes (List et al. 2005). Lenalidomide is known to reduce NF-κB activity and suppress pro-inflammatory cytokines such as TNF-α and IL-6 (Li et al. 2022), both of which are biologically relevant to PAD pathogenesis. However, its known adverse effects, including thrombotic risk (Hirsh 2007), underscore the need for careful evaluation of dosing, patient selection, and vascular safety before considering clinical application in PAD. Overall, our results highlight the value of cross-population MR for identifying both shared and population-specific drug targets while informing rational prioritization for experimental and translational follow-up.

One advantage of this study is the inclusion of multiple populations, which allowed us to investigate both pan-population and population-specific effects on CVD risk. This diversity not only enhances the generalizability of our findings but also provides valuable insights into genetic heterogeneity that might otherwise be overlooked in single-population analyses (Munafò and Davey Smith 2018; Kamstrup and Nordestgaard 2016). Notably, MR signals exhibiting heterogeneity were retained in our candidate list, as such heterogeneity may reflect population-specific linkage disequilibrium structure and allele frequency differences affecting genetic instrument tagging, as well as population-specific environmental and lifestyle factors that interact with genetic variation to influence protein regulation and cardiovascular risk (Zheng et al. 2017).

Another strength of this study is that we utilized a two-sample MR design to estimate potential causal relationships between protein levels in plasma and CVD risk. This methodology leverages large-scale genetic association summary data, where variant-exposure and variant-outcome associations are derived from two independent GWAS. The extensive sample sizes inherent in this approach enable the detection of small causal effect sizes linked to common genetic variants (Wu et al. 2020; Gage et al. 2017). Furthermore, this design enhances statistical power and flexibility in the selection of exposure and outcome variables. It also helps reduce biases from unmeasured confounding and reverse causality (Ghoneim et al. 2020; Lawlor 2016).

Our study also has several limitations that need to be acknowledged to appropriately interpret our findings. First, for IHD, limited GWAS have been conducted in non-European populations, so our study only includes European participants. Second, for certain diseases of interest (AF and HF), we could not use GWAS summary statistics involving UKB data for European and African populations. For example, Nielsen et al. conducted a GWAS of over 1,000,000 subjects of European population, including 1367 AF cases and 29,835 controls from six contributing studies, including UKB (Nielsen et al. 2018). Roselli et al. conducted a GWAS of AF involving 1307 African American cases and 7,660 controls, including UKB participants (Roselli et al. 2018). For HF, the GBMI has collated a large-scale African genetic dataset comprising 1367 HF cases and 29,835 controls, which includes samples from UKB (Zhou et al. 2022). Due to concerns regarding sample overlap between the pQTL studies and these GWAS datasets, which may bias two-sample MR estimates, these datasets were excluded from our study (Panahi et al. 2019). Third, the inclusion of non-specific pQTLs (genetic variants that are associated with the expression levels of multiple proteins, rather than being specific to a single protein) as instruments in our study may influence our MR findings through pleiotropy. For instance, pleiotropic pQTLs within the ABO region have been associated with multiple proteins (Sun et al. 2018). In this study, we found that ABO might have causal relationships with CAD, IHD, and PAD. The pathogenic pathways underlying CVDs are complex and interrelated (Dabic et al. 2023; Fagiry et al. 2021), requiring cautious interpretation and further validation of these results. Finally, substantial heterogeneity was observed in effect estimates across populations for several identified proteins. In addition to distinct genetic effects across populations, this heterogeneity could also result from differences in methodologies employed by the numerous collaborating studies.

In summary, in this study, we identified population-specific and pan-population protein-CVD associations and prioritized 14 protein-CVD pairs as potential drug targets for CVD prevention and treatment. Additional functional validation for the novel proteins uncovered in our analysis is needed to clarify their exact roles in CVD pathophysiology and pharmacological interventions. Overall, our findings underscore the power of proteome-wide MR analysis across populations and emphasize the need to expand research efforts in underrepresented populations to enhance the generalizability and impact of these discoveries.

## Fundings

Lang Wu is supported by National Human Genome Research Institute/National Institute on Minority Health and Health Disparities (NHGRI/NIMHD) U54 HG013243 and National Cancer Institute R01CA263494 and U01CA293883. Hua Zhong was partially supported by award number U24DK132746-01, UCLA LIFT-UP (Leveraging Institutional support for Talented, Upcoming Physicians and/or Scientists). Hua Zhong is partially supported under award number U24 DK132740, LAUNCHED Pilot and Feasibility Grant.

## Electronic Supplementary Material

Below is the link to the electronic supplementary material.

Genetic instruments used in the proteome-wide Mendelian randomization analyses across cardiovascular outcomes and populations.

Summary of the genome-wide association study datasets used for the cardiovascular disease outcomes.

Significant proteome-wide Mendelian randomization associations and sensitivity analyses across cardiovascular outcomes and populations.

Ingenuity Pathway Analysis of the candidate proteins associated with cardiovascular disease outcomes.

Cross-ancestry meta-analysis of proteins associated with cardiovascular disease outcomes.

DrugBank annotations for the proteins associated with cardiovascular disease outcomes.

Prioritized drug-repurposing opportunities with clinical trial information for proteins associated with cardiovascular disease outcomes.

## Data Availability

The pQTL GWAS summary statistics from UKB can be accessed at http://ukb-ppp.gwas.eu). The GWAS summary statistics for CVD generated using BBJ are available from the Japanese ENcyclopedia of GEnetic associations by Riken (JENGER) website (http://jenger.riken.jp/en/result). The GWAS summary statistics from FinnGen are accessible from https://storage.googleapis.com/finngen-public-data-r9/summary_stats/finngen_R9_I9_AF.gz and https://storage.googleapis.com/finngen-public-data-r9/summary_stats/finngen_R9_I9_IHD.gz. The GBMI GWAS summary statistics used were obtained on the GBMI website (https://www.globalbiobankmeta.org/). The GWAS summary statistics from MVP can be downloaded from dbGaP (accession pha004958.1, pha004959.1, pha005193.1, pha005195.1, and pha005240.1). Further information is available from the corresponding author upon reasonable request.
